# METTL3 affects FLT3-ITD+ acute myeloid leukemia by mediating autophagy by regulating PSMA3-AS1 stability

**DOI:** 10.1080/15384101.2023.2204770

**Published:** 2023-04-23

**Authors:** Shenghao Wu, Shanshan Weng, Wenjin Zhou, Yuemiao Chen, Zhen Liu

**Affiliations:** Department of Hematology, The Dingli Clinical Institute of Wenzhou Medical University (The Second Affiliated Hospital of Shanghai University , Wenzhou Central Hospital), Wenzhou, Zhejiang Province, China

**Keywords:** Acute myeloid leukemia (AML), FLT3-ITD, PSMA3-AS1, N6-methyladenosine, autophagy

## Abstract

The study was designed to explore the role of PSMA3-AS1 in initiation and progression of acute myeloid leukemia (AML) and investigate its action mechanism. Expression of PSMA3-AS1, miR-20a-5p and ATG16L1 both *in vitro* and *in vivo* was measured by qRT-PCR. The expression of protein was detected by western blot assay. Edu staining and flow cytometry were utilized to measure cell proliferation and apoptosis. Potential target was predicted by bioinformatics and was verified by dual-luciferase report gene assay and RNA pull down assay. QRT-PCR was used to quantify autophagy (LC3, Beclin1, P62) related genes. The m6A modification test is used to verify the effect of METTL3 on PSMA3-AS1. Tumor model was used to identify the effect of PSMA3-AS1 on tumor growth *in vivo*, and immunohistochemistry was applied to detect expression of ki67 and TUNEL. The results indicate that PSMA3-AS1 was upregulated in FLT3-ITD+ AML patients. Si-PSMA3-AS1 could inhibit the proliferation, autophagy and promote the apoptosis in MV4-11 and Molm13 cells. METTL3 could enhance the PSMA3-AS1 RNA stability. In addition, this study revealed that PSMA3-AS1 affected FLT3-ITD+ AML by targeting expression of miR-20a-5p, and miR-20a-5p further modulated expression of ATG16L1, an mRNA that down-regulated in AML, to affect disease advancement. PSMA3-AS1 could promote FLT3-ITD+ AML progression by regulating the level of autophagy through miR-20a-5p/ATG16L1 pathway. In addition, the increase of PSMA3-AS1 may be caused by the involvement of METTL3 in regulating its stability. This discovery will provide new horizons for early screening and targeted therapy of FLT3-ITD+ AML.

## Introduction

Acute myeloid leukemia (AML) is a hematological malignancy originating from hematopoietic stem cells [1]. Nearly half of acute myeloid leukemias have genetic abnormalities, and genetic abnormalities endow leukemia cells with different biological characteristics, such as cell proliferation, tissue infiltration and invasion, which manifest as uncontrolled proliferation and blocked differentiation of immature cells in the hematopoietic system lead to leukemia [2]. The development of molecular biology techniques has led to the emergence of new branches in the field of genetics, and has contributed to the discovery of many characteristic molecular and genetic abnormalities of AML at the microscopic level (including various fusion genes, genetic mutations, and genetic modifications) [3]. The molecular changes of AML are one of the most important factors affecting the prognosis of patients [4]. At present, a series of molecular tests have been included in the classification and prognosis analysis of AML. NPM1, FLT3-ITD, CEBPA, MLL, RUNX1, EZH2, etc. [5]. The integrated classification standard improves the prognosis stratified diagnosis and treatment strategy of leukemia patients. Cells with different genetic abnormalities have different sensitivities to chemotherapy and targeted drugs, making the remission rate, relapse rate and survival rate of different types of acute myeloid leukemia significantly different. Among them, NPM1 and CEBPA mutations suggest good prognosis, and FLT3-ITD, RUNX1, EZH2 mutations are molecular biomarkers of poor prognosis, and the prognosis is worse when FLT3-ITD gene mutations coexist. At present, a variety of FLT3 inhibitors (FLT3i), including the first generation of mitdolitoline and the second generation of giletinib, krononib and quizatinib, have entered clinical trials. Although clinical data show that this kind of drug has some efficacy, studies have shown that the efficacy of the second generation of FLT3i is not lasting and often develops into recurrent FLT3-ITD AML within a few months [3,6]. Therefore, seeking new treatment strategies is still the research direction of FLT3-ITD AML.

RNA methylation mainly regulates gene expression at the post-transcriptional level and is considered to be another layer of epigenetic regulation similar to DNA methylation and histone modification [7]. In recent years, various molecular modifications of RNA (including rRNA, tRNA, snRNA, mRNA and long non-coding RNA) have been discovered, the most common of which is N6-methyladenine (m6A methylation) [8]. m6A methylation is the most prevalent RNA methylation modification in eukaryotes. Studies have found that m6A RNA methylation has important regulatory functions in tissue growth, circadian rhythm, DNA damage response, and sex determination, and is of great significance in tumorigenesis, development, and treatment [9].

METTL3 is involved in the methylation catalytic process as a writer [10]. Current studies have shown that METTL3 can participate in various tumor processes including AML [11]. Among them, METTL3 can affect tumor progression by regulating the stability of lncRNA [12,13]. As a kind of long non-coding RNA, PSMA3-AS1 has been shown to increase its expression and participate in the promotion of tumor progression in various tumor studies. However, the expression of PSMA3-AS1 in FLT3-ITD AML has not been paid attention to, and whether its regulatory relationship with METTL3 can affect the progression of FLT3-ITD AML is unclear. Therefore, in this study, the regulatory role of METTL3 and PSMA3-AS1 in the disease process was verified by bioinformatics combined with in vitro and in vivo experiments, which provided the basis for the development of targeted drugs for FLT3-ITD AML.

## Materials and methods

### Ethical statement

All subjects in this study signed written informed consent which was protocolled according to the guidelines. All experiments of this study were supported by the Clinical Research Ethics Committee of Wenzhou Central Hospital.

### Clinical sample

The samples were collected from 60 patients who were diagnosed as AML (including 30 FLT3-ITD+ AML patients, 30 FLT3-ITD- AML patients) and 30 healthy donors. Among those participants, the gender ratio approached 1:1 and the ages of them were between 30 and 60. All materials were admitted to Wenzhou Central Hospital. The samples were extracted out of patients and volunteers by operating and then were stored at−80°C for use.

### Cell culture and transfection

Human cell line Molm-13 (FLT3-ITD+ cell line), MV4–11 (FLT3-ITD+ cell line), THP-1 (FLT-ITD- cell line) and U937 (FLT-ITD- cell line) were obtained from ATCC (Manassas, VA, USA) and was cultured in RPMI 1640. Medium supplemented was with 10% FBS (fetal bovine serum) storing at 37°C with 5% CO_2_.

The cDNA of PSMA3-AS1 was synthesized according to the sequence obtained from lncBase database, and then cloned into pcDNA3.1 plasmid using TA Cloning™ Kit (Thermo Fisher Scientific). PcDNA3.1 plasmid vectors, pcDNA3.1-PSMA3-AS1 (oe-PSMA3-AS1), siRNA- PSMA3-AS1 (si-PSMA3-AS1), pcDNA3.1-ATG16L1 was obtained from Genechem (Shanghai, China). MiR-20a-5p mimics and miRNA negative control were synthesized by RiBoBio (Guangzhou, China). Transfection of blank plasmid was applied to the control group (Ctrls). Cells were transiently transfected by Lipofectamine 2000 reagent (Invitrogen) on the basis of the manufacturer’s protocol. After being transfected for 24 h, cells were obtained and used for experiments.

### Isolation and culture of primary AML cells

Mononuclear cells were isolated by centrifugation through Ficoll-Hypaque from the peripheral blood derived from FLT3-ITD-positive AML patients at diagnosis with white blood cell count of 149, 000/μl with 80% blasts or 94, 800/μl with 81% blasts (Case 1 or 2, respectively). Cryopreserved cells were thawed and cultured for 1 day in IMDM with 10% FBS [14].

### Expression profile analysis of lncRnas

The lncRNA chip analysis data came from the GEO database (GSE103828). The 10 samples got analyzed using lncRNA chips. In the study presented here, 5 pairs of AML patients and iron deficiency anemia (IDA) controls were screened by microarray. Labeled RNAs were scanned and acquired by employing an Agilent -079,487 Arraystar Human LncRNA microarray V4 (Agilent Technologies, USA), and array images were analyzed by Agilent Feature Extraction software (version 11.0.1.1, USA). The differentially expressed lncRNAs were selected according to the fold-change cutoff (fold change ≥2) and *P*-value <0.05.

### The Cancer Genome Atlas (TCGA) database analysis

RNAseq data (level 3) of 151 AML samples (38 treated as controls and 113 untreated as study) were obtained from The Cancer Genome Atlas (TCGA) dataset (https://portal.gdc.com) and corresponding clinical information. m6A-related genes were derived from Juan Xu et al.‘s study on the molecular characterization and clinical significance of m6A regulators across 33 cancer types.

The above results were achieved with the R (v4.0.3) packages ggplot2 and pheatmap.

### RNA isolation and quantitative real-time PCR (Qrt-PCR)

Total RNA extraction from purified cell lines was performed by using TRIzol (Life Technologies, Gaithersburg, MD, USA). 1 μg of RNA was treated with DNase I prior and then reverse transcription into cDNA. qRT-PCR assays functioned by using Platinum® SYBR® Green qPCR SuperMix (Life Technologies) which made housekeeping gene GAPDH or U6 the internal control. The 2^−ΔΔCt^ (comparative threshold cycle) method was employed to identify the relative quantification of gene expression levels. RiboBio (Guangzhou, China) was employed to synthesize the premier sequences, and the results were shown in Table S1.

### Western blot

Cell lysates were disposed by a detergent buffer. Protein concentrations were measured with the BCA Protein Assay on the basis of the manufacturer’s manual (Beyotime Institute of Biotechnology, Shanghai, China). Equal amounts (80 μg) of protein were isolated by 12% SDS-PAGE gel electrophoresis and were transferred to polyvinylidene difluoride membranes (PVDF, Millipore, Billerica, MA). After being blocked by 5% nonfat dry milk (NFDM), membranes achieved incubated overnight at 4°C with a 1:1000 dilution of anti-ATG16L1 (ab187671, Abcam, Cambridge, MA, USA) or GAPDH (ab8245, Abcam). After additional incubation with a 1:1000 dilution of a goat anti-mouse horseradish peroxidase-linked antibody (ab6785) for 1 h, protein bands on the blots were measured by the use of Image J software (Bio-Rad Laboratories, USA).

### MTT assay

The cell proliferation of each treatment group was determined by measuring the amount of MTT reduced to formaldehyde. MTT reagent was then supplemented, followed by cell incubation for 3 h. The solution was decanted, and 100 μL DMSO was added for dissolution of purple formazan crystals. The absorbance of the resulting solution was measured at 570 nm with a microplate (Bio, USA).

### Edu staining

To measure proliferation potential of cells, cells were subjected to the Cell-Light TM EdU imaging detecting kit (RiboBio, Shanghai, China). Briefly, each well of a 96-well plate contained cells (1 × 10^5^/well). After being cultured with 10 mM EdU for 2 h, all the Cells were fixed with 4% paraformaldehyde, followed by permeabilization with 0.2% Triton X-100 and stained by Apollo solution for 30 min in the dark. Subsequently, cell nuclei got stained by DAPI solution.

### Cell apoptosis analysis

Cells were harvested from different groups for apoptosis analysis after double staining of the cells with Annexin-V and Propidiumiodide (PI) via using Annexin-V-FLOUS staining kit in the basis of the manufacturer’s protocol (Roche, Mannheim, Germany).

### Dual luciferase activity assay

Subcloned into luciferase reporter psiCHECK2 (Promega, Madison, WI). On 96-well plates, cells were seeded in the density of 3 × 10^4^ cells per well for 24 h in triplicate before transfection and then transfected with wild-type or mutated reporter vectors, and miRNA mimics or negative control. Lysates were harvested after being transfected for 24 h.

### RNA stability assays

MV4–11 cells were treated with actinomycin D at a final concentration of 5 μg/ml, and the experiment was divided into control group and METTL3 knockout group. After 0, 2 and 4 hours of treatment, cells in each group were collected for RNA isolation. Then, the expression of PSM3-AS1 in each treatment group was detected by qRT-PCR.

### RIP assay

RIP was employed by using a Magna RNA-binding protein immunoprecipitation kit (Millipore, Billerica, MA, USA) on the basis of the manufacturer’s instructions. MV4–11 cell lysates containing PSMA3-AS1, ATG16L1 and miR-20a-5p got prepared and incubated with anti-argonaute2 (Ago2) antibody (Millipore). NC was set with Normal mouse IgG (Millipore).

### Animals

Six-week-old nude mice were obtained from Shanghai Lab. Animal Research Center (Shanghai, China). A total of eighteen mice were randomly divided into three groups, and were injected with serum-free cell suspensions of MV4–11 cells that transfected with vector (as negative control, named Ctrls) or si-METTL3, respectively. Tumor size was observed every 6 days for 42 days and was calculated by the following formula: 1/2×L^2^×W (L, length (mm); W, width (mm) of the tumor). When the experiment approached termination (the 42st day), the tumor in the body of each mouse was excised and was weighted.

### Immunohistochemistry (IHC)

Longitudinal sections of tumor specimens were fixed in 10% formalin, embedded in paraffin, and then completed by deparaffinizing with xylene and hydrating with an ethanol gradient. After being successively incubated with antigen retrieval solution (Shunbai, China) and 3% H_2_O_2_ for 30 min, the slides were washed with water and cultured with the primary antibody anti-ki67 (1:100) and anti-TUNEL (Sevier, China) overnight at 4°C. Nonimmunized serum was utilized to dispose negative controls with discarding primary antibody. The next day, secondary antibody (Beijing Biosynthesis Biotechnology Co. Ltd.; Beijing, China) was used to rinse and incubate slides, which then was followed by 3, 3′-diaminobenzidine (DAB) and hematoxylin staining, respectively. Image-J software was performed to analyze the results of DAB staining.

### Statistical analysis

All the data were analyzed by using GraphPad Prism 6.0, and they were presented as the mean ± SD (standard deviation). The *in vitro* experiments were performed three times. Difference between two groups were identified by Students’ T-test and to measure differences among three groups or more, one-way ANOVA was employed. *P*-values less than 0.05 was considered as a statistically significant difference.

## Results

### The PSMA3-AS1 was high expressed in FLT3-ITD+ AML

**Figure 1.**
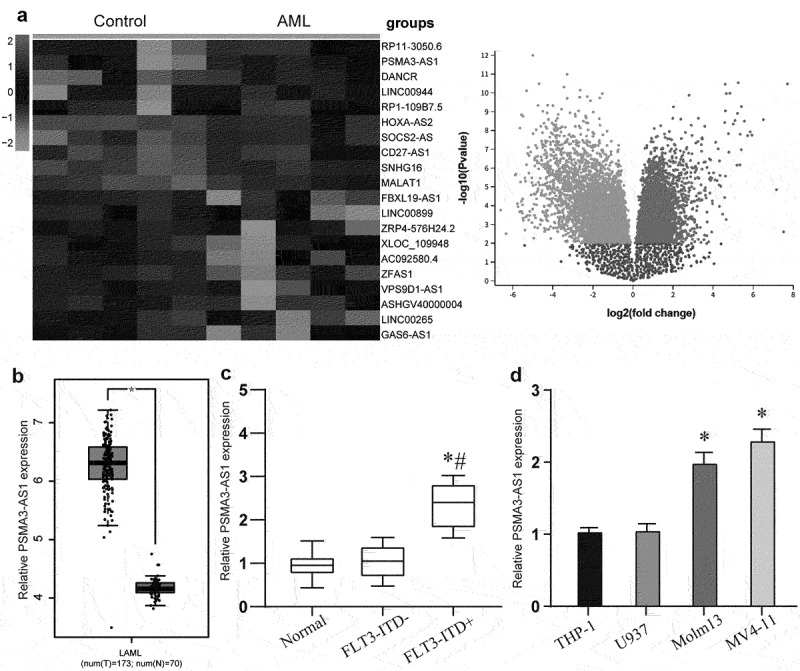
PSMA3-AS1 was up-regulated in FLT3-ITD+ AML. (a) The heat map and volcano map showed the top 10 most increased and 10 decreased lncRnas in AML patients as compared to that in the control samples analyzed by lncRnas Arraystar Chip from GSE103828. (b) Relative expression of PSMA3-AS1 was measured in AML of TCGA database. (c) Relative expression of PSMA3-AS1 was detected in AML patients by Qrt-PCR. (D) Qrt-PCR was performed to determine expression of PSMA3-AS1 in FLT3-ITD+ or FLT3-ITD- cells. *P < .05.

To explore differentially expressed lncRNAs between AML patients and healthy subjects, we performed lncRNA microarray assay analysis on data from GSE103828. The data included five control samples and five samples from AML patients. The differently expressed lncRNAs were identified to base on the fold change more than 2. Heatmap showed 20 most abnormal expressed lncRNAs, including 10 downregulated lncRNAs and 10 upregulated lncRNAs (Figure 1a). Further analysis of PSMA3-AS1 through the TCGA database found that it was significantly overexpressed in AML (Figure 1b) (*P* < 0.05). Furthermore, our clinical samples showed that the expression level of PSMA3-AS1 was higher in FLT3-ITD+ patients than in FLT3-ITD- patients and controls (Figure 1c, *P* < 0.05). Compared with FLT3-ITD- THP-1 and U937 cells, the expression of PSMA3-AS1 was significantly increased in FLT3-ITD+ Molm-13 and MV4–11 cells (Figure 1d, *P* < 0.05). Finally, we verified that the expression of PSMA3-AS1 was significantly elevated in patient-derived primary AML cells (Supplementary Figure S1A). These evidences indicate that PASM3-AS1 was highly expressed in FLT3-ITD+ patients and in FLT3-ITD+ cell lines (Molm-13, MV4–11).

### LncRNA PSMA3-AS1 promoted the proliferation and inhibited apoptosis of MV4–11 cells

**Figure 2.**
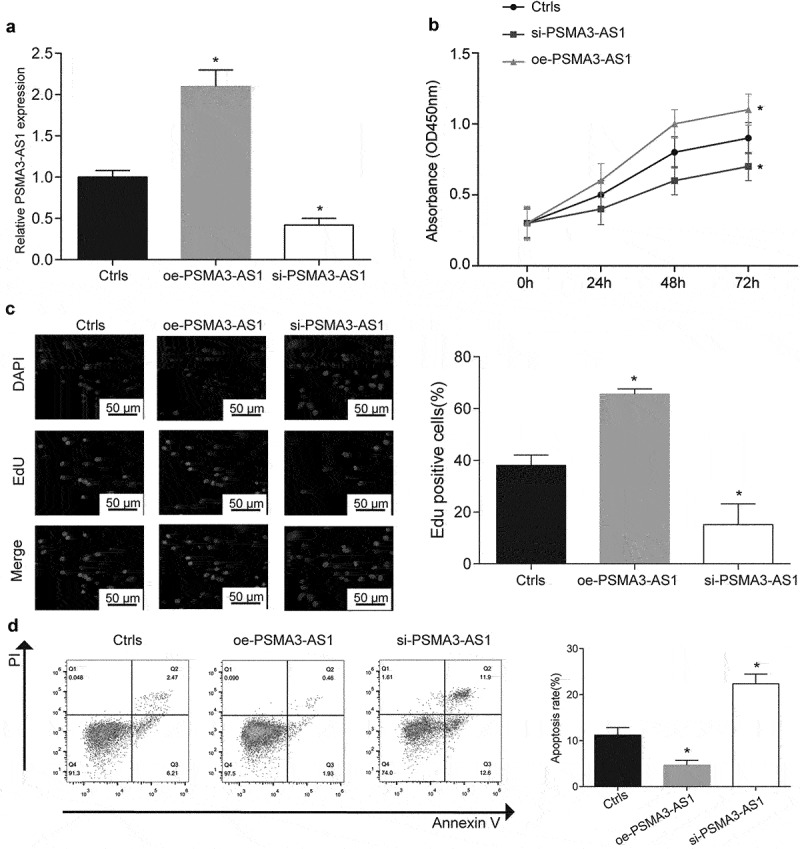
The influence of different expression levels of PSMA3-AS1 in MV4–11 cells proliferation and apoptosis. (a) Qrt-PCR was conducted to determine the transfection efficiency of PSMA3-AS1. (b) MTT was used to detect cell viability in different treatment groups. (c) EdU staining was used to test the cell viability in the different groups. Scale bars represent 50 μm. (d) The effect of PSMA3-AS1 on cells apoptosis was investigated by using flow cytometry. *P < .05.

To further explore the action mechanism of PSMA3-AS1 expression on FLT3-ITD+ AML, we investigated the effects of PSMA3-AS1 expression on cell proliferation and apoptosis by using MV4–11 cells. QRT-PCR assay identified that PSMA3-AS1 expression was significantly up-regulated in MV4–11 cells transfected with oe-PSMA3-AS1, while its expression was reduced in si-PSMA3-AS1 group compared with Ctrls group (Figure 2a, *P* < 0.05). The results of MTT analysis showed that oe-PSMA3-AS1 could significantly promote cell proliferation, while inhibiting its expression showed the opposite result. This was also confirmed in Edu staining (Figure 2b,c *P* < 0.05). In addition, apoptosis rate of cells was investigated by flow cytometry, and the result revealed that si-PSMA3-AS1 was involved in MV4–11 cell apoptosis. Briefly, si-PSMA3-AS1 elevated apoptosis rate of MV4–11 cell, oe-PSMA3-AS1 inhibited cells apoptosis (Figure 2d, *P* < 0.05). Furthermore, we validated the function of PSMA3-AS1 in Molm13 cells. Our results show that overexpression of PSMA3-AS1 can promote the proliferation of Molm13 and inhibit its apoptosis (Supplementary Figure S2A-D).

### Autophagy and the role of ATG16L1 in the regulation of MV4–11 cell progression by PSMA3-AS1

**Figure 3.**
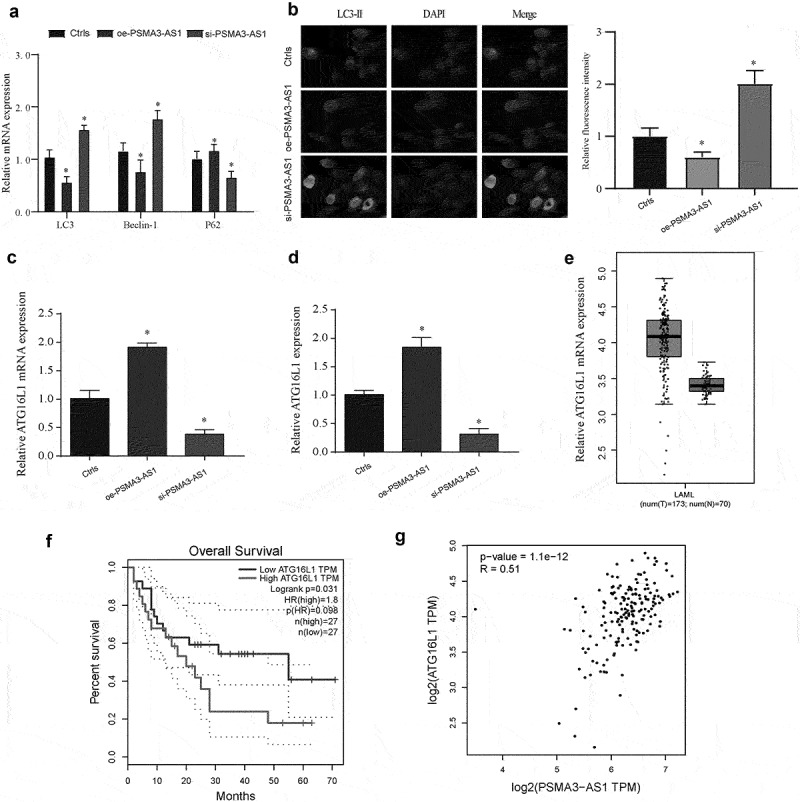
Autophagy and the role of ATG16L1 in the regulation of MV4–11 cell progression by PSMA3-AS1. (a) The regulation of PSMA3-AS1 on the expression of autophagy markers was detected by Qrt-PCR. (b) Immunofluorescence detection of autophagosome expression in different treatment groups. (c–d) Expression of ATG16L1 Mrna (c) and protein (d) after different treatments of PSMA3-AS1. (e) Relative expression of ATG16L1 was measured in AML of TCGA database. (f) Analysis of the prognosis of AML with different expression of ATG16L1 by KM curve. (g) Pearson correlation analysis of the correlation between PSMA3-AS1 and ATG16L1 in AML. *P < 0.05.

Current studies had shown that autophagy plays an important role in AML progression, but its role in FLT3-ITD+ AML and its relationship with PSMA3-AS1 have not been demonstrated [15–17]. Therefore, we detected the expression of autophagy markers in different treatment groups and found that si-PSMA3-AS1 could significantly inhibit the level of autophagy while oe-PSMA3-AS1 significantly promoted this process (Figure 3a, *P* < 0.05). We further demonstrated this phenomenon by labeling autophagosomes with LC3 (Figure 3b, *P* < 0.05). Similarly, this phenomenon was confirmed in Molm13 cells (Supplementary Figure S2E-F). These results suggest that autophagy is involved in the regulation of FLT3-ITD+ cells by PSMA3-AS1. A recent study showed that ATG16L1 plays an important regulatory role in AML autophagy [18], so we detected its expression and found that compared with the ctrls group, overexpression of PSMA3-AS1 could significantly promote the mRNA and protein expression of ATG16L1 (Figure 3c,d, *P* < 0.05). We further verified this expression using TCGA data and found that high ATG16L1 expression was significantly associated with poor prognosis (Figure 3e,f, *P* < 0.05). Finally, we found a positive correlation between PSMA3-AS1 and ATG16L1 (Figure 3g, *P* < 0.05). This regulatory mechanism has attracted the interest of researchers.

### MiR-20a-5p was a regulator of PSMA3-AS1 and ATG16L1

**Figure 4.**
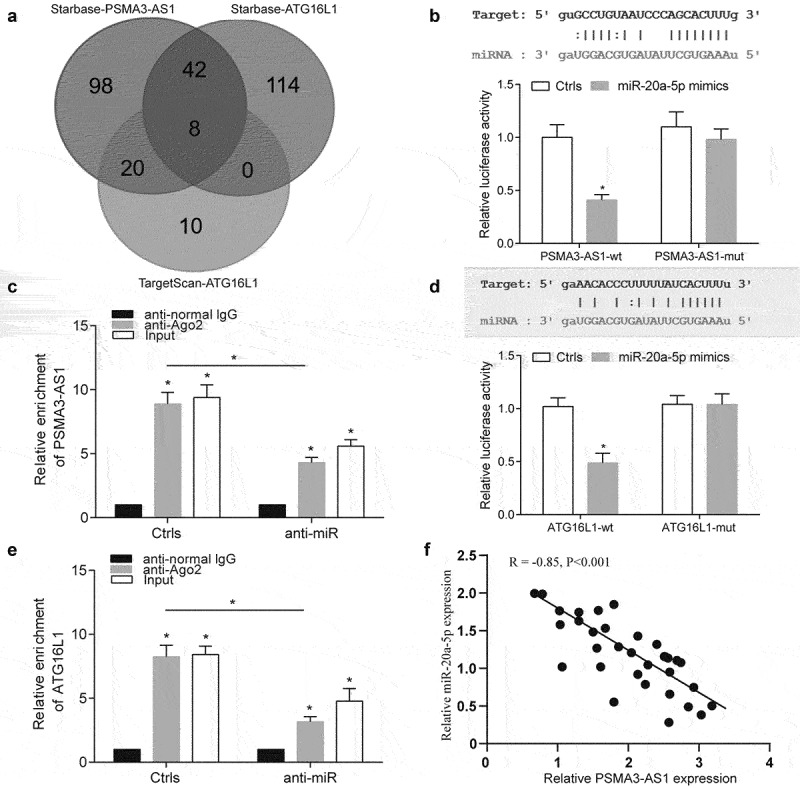
MiR-20a-5p was a regulator of PSMA3-AS1 and ATG16L1. (a) Draw Venn diagram for targeted miRNA screening of PSMA3-AS1 and ATG16L1. (b) Dual luciferase assay verified that miR-20a-5p was direct target of PSMA3-AS1. (c) RIP was used to detect the enrichment of PSMA3-AS1 after miR-20a-5p treatment. (d) Dual luciferase assay verified that ATG16L1 was direct target of miR-20a-5p. (e) RIP assay was used to detect the enrichment of ATG16L1 after miR-20a-5p treatment. (f) Pearson correlation analysis of the correlation between PSMA3-AS1 and miR-20a-5p in FLT3-ITD+ AML. *P < 0.05.

From the above results, we found that ATG16L1 was positively correlated with the expression of PSMA3-AS1, so the competitive adsorption mechanism (ceRNA) attracted the attention of researchers. First, we performed enrichment analysis on the targeted miRNAs of the two databases through Targtscan and Starbase, and found that there were eight common targeted miRNAs (Figure 4a). Further, we decided to first verify the expression of miR-20b-5p through references. Both dual-luciferase reporter gene detection and RIP experiments confirmed our conjecture, and the results showed that PSMA3-AS1 had a targeting relationship with miR-20a-5p (Figure 4b,c *P* < 0.05). This was also confirmed in the targeting of miR-20a-5p and ATG16L1 (Figure 4d,e, *P* < 0.05). This suggests that miR-20a-5p may be an intermediate regulator of PSMA3-AS1 and ATG16L1. We also found that the expression of miR-20a-5p and ATG16L1 in primary AML cells was significantly decreased, and the expression of ATG16L1 was significantly increased (Supplementary Figure S1b and c). Finally, we found a negative correlation between the expression of PSMA3-AS1 and miR-20a-5p in FLT3-ITD+ AML patients, which further verified our conjecture (Figure 4f, *P* < 0.05).

### PSMA3-AS1 affects the proliferation and autophagy of FLT3-ITD+ cells by regulating the expression of ATG16L1 through miR-20a-5p

**Figure 5.**
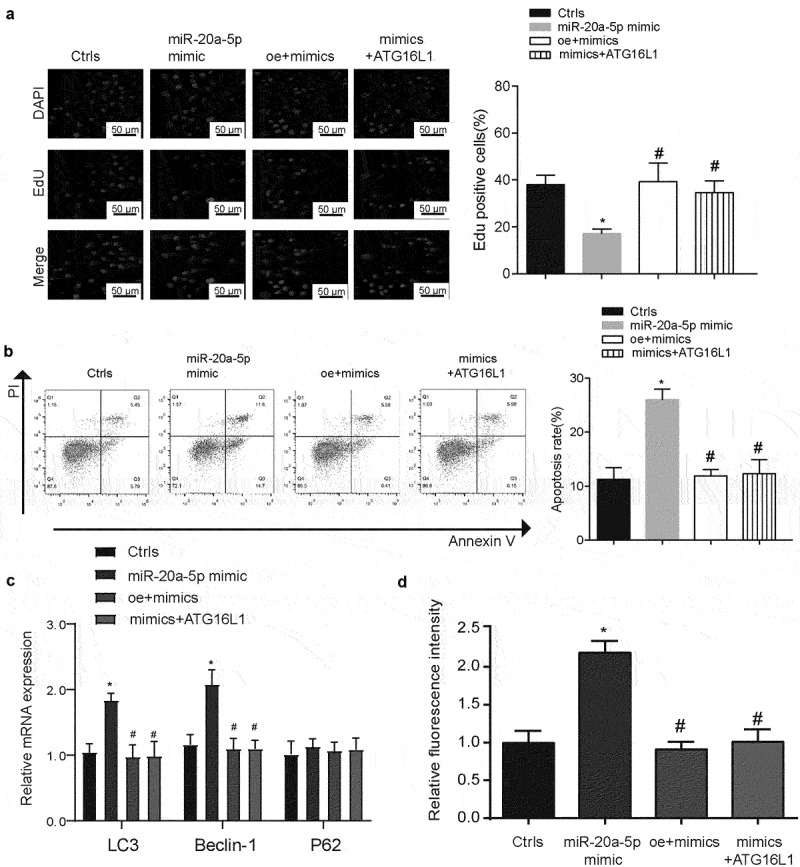
PSMA3-AS1 affects the proliferation and autophagy of FLT3-ITD+ cells by regulating the expression of ATG16L1 through miR-20a-5p. (a) EdU staining was used to test the cell viability in the different groups. Scale bars represent 50 μm. (b) The effect of PSMA3-AS1, miR-20a-5p and ATG16L1 on cells apoptosis was investigated by using flow cytometry. (c) The regulation of PSMA3-AS1, miR-20a-5p and ATG16L1 on the expression of autophagy markers was detected by Qrt-PCR. (d) Immunofluorescence detection of autophagosome expression in different treatment groups. *P < 0.05.

To determine the modulated capacity of miR-20a-5p in progression of FLT3-ITD+ AML, this research explored the effects of miR-20a-5p on proliferation and apoptosis of MV4–11 cells. MV4–11 cells were co-transfected with Lipofectamine 2000 (Ctrls), miR-20a-5p mimics, oe-PSMA3-AS1+miR-20a-5p-mimics (oe+mimics) or ATG16L1+miR-20a-5p-mimics (Supplementary Figure S3). As shown in Figure 5a, Edu staining assay found that Edu positive cell rate decreased in miR-20a-5p mimics group and the effect was reversed by PSMA3-AS1 or ATG16L1 (*P* < 0.05). Cell apoptosis was detected using flow cytometry. Results showed that miR-20a-5p significantly promoted apoptosis ability of MV4–11 cells (Figure 5b, *P* < 0.05). Whereas, there were no statistical significance between functional oe-PSMA3-AS1+miR-20a-5p-mimics (oe+mimics) or ATG16L1+miR-20a-5p-mimics groups and Ctrls group (*P* > 0.05). This effect also applies to the detection of autophagy levels (Figure 5c,d, *P* < 0.05). We also verified this trend in Molm13 cells. We found that their results tended to be similar (Supplementary Figure S4). These data suggest that PSMA3-A1 regulates FLT3-ITD+ AML progression and autophagy levels via miR-20a-5p/ATG16L1.

### METTL3 affects PSMA3-AS1 RNA stability

Recently, cutting-edge studies have shown that m6A modifications in mRNAs and lncRNAs are extremely extensive and functionally regulate the eukaryotic transcriptome, affecting RNA splicing, export, localization, translation and stability. First, we obtained the AML assay microarray data from the TCGA database. We found differential expression of m6A modifier genes (METTL3 and RBM15) (Supplementary Figure S5). These results suggest that m6A modification is involved in the regulation of AML progression. However, METTL3 was brought to our attention as an up-regulated gene, as this might explain the increase in PSMA3-AS1.

**Figure 6.**
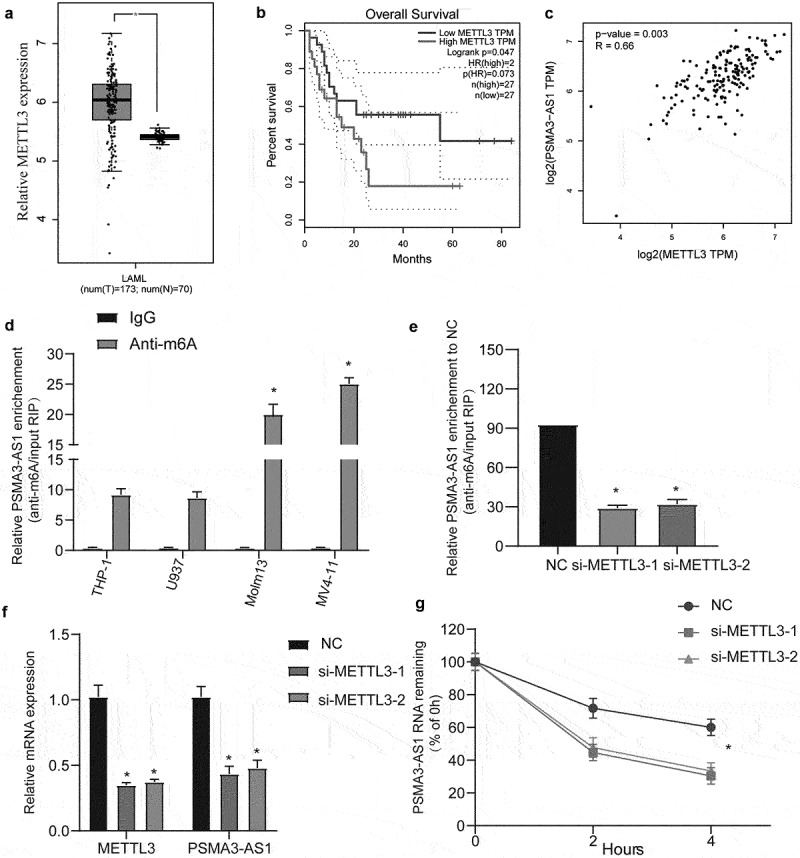
METTL3 affects PSMA3-AS1 RNA stability. (a) Relative expression of PSMA3-AS1 was measured in AML of TCGA database. (b) Analysis of the prognosis of AML with different expression of METTL3 by KM curve. (c) Pearson correlation analysis of the correlation between PSMA3-AS1 and METTL3 in AML. (d) The M6A methylation level of PSMA3-AS1 in FLT3-ITD- cells (THP-1 and U937) and FLT3-ITD+ cells (Molm-13 and MV4–11) was determined by MeRIP-Qpcr analysis. (e) The change of m6A-modified PSMA3-AS1 increased when METTL3 interfered with the expression. (f). QRT-PCR detects the expression of PSMA3-AS1 and METTL3 after si-METTL3 treatment. (g) Compared with the control, the stability of PSMA3-AS1 RNA was reduced in MV4–11 cells with knockout of METTL3 gene. *P < 0.05.

The TCGA database first found that METTL3 expression was significantly elevated and correlated with poor prognosis (Figure 6a,b, *P* < 0.05). Furthermore, correlation analysis showed that METTL3 was positively correlated with PSMA3-AS1 expression (*R* = 0.66, *P* = 0.002) (Figure 6c). Next, we performed methylated RNA immunoprecipitation analysis on FLT3-ITD- cells (THP-1 and U937) and FLT3-ITD+ cells (Molm-13 and MV4–11) (Figure 6d, *P* < 0.05). The results showed that m6A levels were significantly elevated in Molm-13 and MV4–11 cells. We found that downregulation of METLL3 was able to inhibit the enrichment and expression of PSMA3-AS1 (Figure 6e,f, *P* < 0.05). Finally, we measured the shortened half-life and poor stability of PSMA3-AS1 after METTL3 silencing after blocking new RNA synthesis with actinomycin D (Figure 6g, *P* < 0.05). These findings may explain the significant upregulation of PSMA3-AS1 in FLT3-ITD+ AML.

### In vitro and in vivo assays to verify the regulatory effect of METTL3 on FLT3-ITD+ AML progression

**Figure 7.**
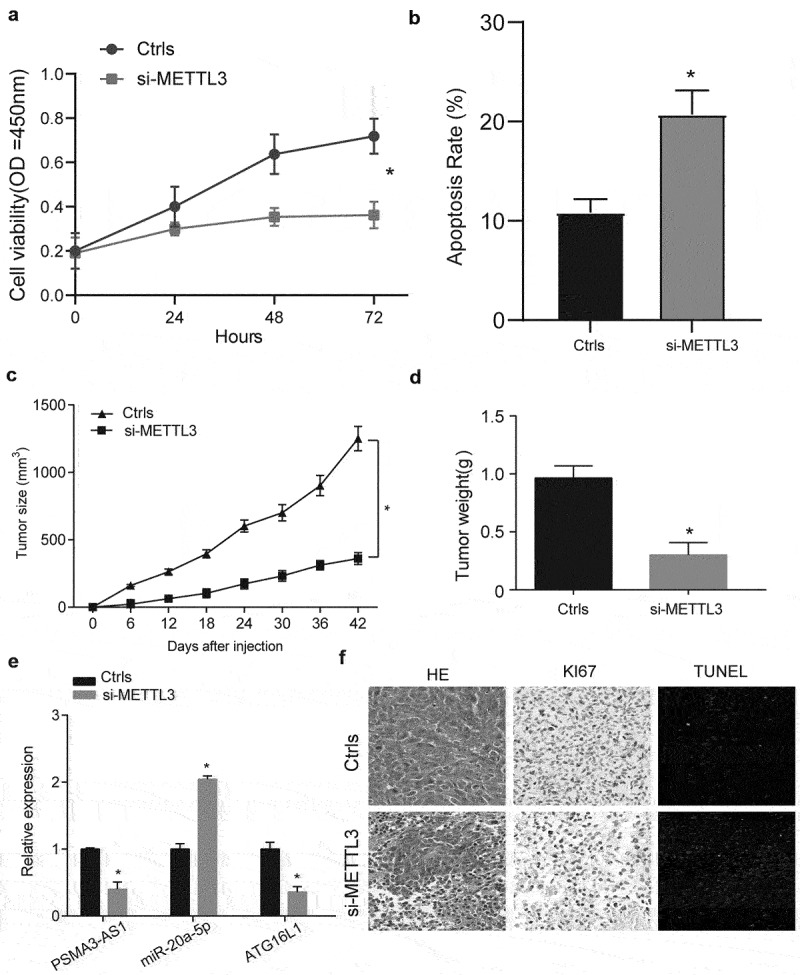
In vitro and in vivo assays to verify the regulatory effect of METTL3 on FLT3-ITD+ AML progression. (a) MTT was used to detect cell viability in different treatment groups. (b) The effect of METTL3 on cells apoptosis was investigated by using flow cytometry. (c) The tumor size was measured every 6 days for 42 days, and tumor volume was calculated. (d) Tumors were excided from nude mice who were injected with MV4–11 cells transfected with ctrl or si-METTL3. The tumor weight were measured. (e) Qrt-PCR assay was used to examine expression of PSMA3-AS1, miR-20a-5p and ATG16L1 for different transfected groups in vivo. (f) Expression of Ki67 and TUNEL in each group was detected by immunohistochemical staining. *P < 0.05.

First, we verified that inhibition of METTL3 could inhibit cell proliferation and promote apoptosis by MTT and flow cytometry (Figure 7a,b, *P* < 0.05). From the view of growth curve of tumor size, we apparently found that knockdown METTL3 inhibited tumor growth compared with Ctrls group (Figure 7c, *P* < 0.05). Tumors were weighted after excised, and we observed that tumors’ weight in si-METTL3 group was evidently lower than that in Ctrls group (Figure 7d, *P* < 0.05). Afterwards, qRT-PCR was performed to quantitate the expression level of PSMA3-AS1, miR-20a-5p and ATG16L1, as shown in Figure 7e. The results showed that si-METTL3 inhibited the expression of PASM3-AS1 and ATG16L1 and promoted the expression of miR-20a-5p. Finally, we performed morphological examination on the collected tumor samples and found that si-METTL3 inhibited proliferation and promoted apoptosis in tumor tissue (Figure 7f). These data suggest that METTL3 can regulate disease progression by affecting PSMA3-AS1 stability.

## Discussion

Acute myeloid leukemia (AML) is an aggressive blood cancer with genetic heterogeneity that involves proliferative disorder of the hematopoietic system [19]. The main inducements of AML are uncontrolled proliferation of myeloid progenitor cells 26376137, abnormal differentiation of blast cells, and impaired production of normal blood cells [20]. Adults are easy to suffer AML [21], and this disease is likely to be diagnosed in both infants and older adults [22]. In recent decades, treatment of AML consisted of hematopoietic cell transplantation and intensive induction therapy [23]. Even though initial chemotherapy on 80% of patients who were diagnosed to suffer AML worked, 50% to 60% of them were found to relapse in later 1 to 2 years [24]. Overall, it is urgent to put forward to novel therapy in AML to improve the patients’ survival possibility.

This study is the first to find that PSMA3-AS1 is up-regulated in patients and cells of FLT3-ITD+ AML. In vitro and in vivo experiments confirmed that PSMA3-AS1 can regulate the expression of ATG16L1 through competitive binding to miR-20a-5p and affect the progression of FLT3-ITD+ AML. Furthermore, through in-depth exploration of m6A methylation, we found that METTL3 may be partly responsible for the induction of elevated PSMA3-AS1.

PSMA3-AS1 is a lncRNA transcribed from the antisense strand of PSMA3, and current studies have shown that it can promote lung cancer progression through miR-4504. Another study showed that PSMA3-AS1 can activate the PI3K/AKT pathway to promote cervical cancer proliferation and metastasis through the pathway of miR-378a-3p regulating GALNT3 [25–27]. Our results indicate that PSMA3-AS1 is elevated in FLT3-ITD+ AML tissues and cells and can promote cell proliferation and tumor growth. Furthermore, our results found that PSMA3-AS1 could inhibit autophagy through ATG16L1. In the above studies, it was found that PSMA3-AS1 can activate the PI3K/AKT pathway, which has been shown to inhibit autophagy. This phenomenon is consistent with our findings. In addition, a recent study on AML showed that ATG16L1 could regulate the level of autophagy in FLT3-ITD+ AML, which further confirmed our hypothesis that PSMA3-AS1 regulates disease progression through autophagy [18].

The competitive adsorption mechanism is currently an important mechanism in the regulation of lncRNAs [26], and our results found that PSMA3-AS1 and ATG16L1 have a positive regulatory role, suggesting that miRNAs may play a role in the regulation. We found miR-20a-5p to be their potential targets by screening different databases. This was confirmed in subsequent dual-luciferase reporter and RIP experiments. Several reports targeting miR-20a-5p in AML with reduced expression and inhibition of disease progression corroborate our data. However, we found for the first time that miR-20a-5p can be involved in the regulation of autophagy and disease progression in FLT3-ITD+ AML as a target gene of PSMA3-AS1.

At present, studies have found that the expression of METTL3 is increased in AML, and a large number of studies have found that it can participate in regulating the stability of lncRNA and affect its expression [10,11]. We analyzed the m6A-related genes of AML patients before and after treatment through the data of TCGA database and found that only METTL3 expression was up-regulated and the difference was statistically significant. Therefore, we further verified that it can promote its expression by affecting the stability of PSMA3-AS1. These data provide a plausible explanation for our findings. We also verified the role of METTL3 in AML in this study, which is the first time to our knowledge. Although we have favorably demonstrated our findings *in vitro* and *in vivo*, we still lack more information on follow-up data and diagnostic data to further validate this finding in population samples. So this will be one of the main directions of our future research.

Taken together, we found that PSMA3-AS1 could promote FLT3-ITD+ AML progression by regulating the level of autophagy through the miR-20a-5p/ATG16L1 pathway. In addition, the increase of PSMA3-AS1 may be caused by the involvement of METTL3 in regulating its stability. This discovery will provide new horizons for early screening and targeted therapy of FLT3-ITD+ AML.

## Supplementary Material

Supplemental Material

## Data Availability

The datasets used and/or analyzed during the current study available from the corresponding author on reasonable request.
