# Supplementary material for: METTL3 affects FLT3-ITD+ acute myeloid leukemia by mediating autophagy by regulating PSMA3-AS1 stability
Source: Cell Cycle. 2023 Apr 23;22(10):1232–45. doi: 10.1080/15384101.2023.2204770 (PMC10193868; doi:10.1080/15384101.2023.2204770)
Supplement: Supplemental Material [file KCCY_A_2204770_SM0427.zip › Table S1 (2).docx]

Table S1 Primer Sequences for qRT-PCR

| **Genes** | **Sequences（5’-3’）** |
| --- | --- |
| **PSMA3-AS1** |  |
| Forward primer | AACAGACCATCAGAAGAGAACA |
| Reverse primer | GAACAGAAACCAGAGCCATACA |
| **miR-20a-5p** |  |
| Forward primer | GTAAAGTGCTTATAGTGCAG |
| Reverse primer | GTCGTATCCAGTGCGTGTCG |
| **ATG16L1** |  |
| Forward primer | CAGAGCAGCTACTAAGCGACT |
| Reverse primer | AAAAGGGGAGATTCGGACAGA |
| **METTL3** |  |
| Forward primer | 5’- CCCGCGGATATTCACATGGAACTGCC-3’ |
| Reverse primer | 5’- TGGGGCAGCCATCACAACTGCAAAC-3’ |
| **Beclin1** |  |
| Forward primer | 5’-GGCCAATAAGATGGGTCTGA-3’ |
| Reverse primer | 5’-GCTGCACACAGTCCAGAAAA-3’ |
| **P62** |  |
| Forward primer | 5’-TGTGGAACATGGAGGGAAG-3’ |
| Reverse primer | 5’-TGTGCCTGTGCTGGAACTTTC-3’ |
| **LC3** |  |
| Forward primer | 5’-TTGGTCAAGATCATCCGGC-3’ |
| Reverse primer | 5’-GCTCACCATGCTGTGCTGG-3’ |
| **GAPDH** |  |
| Forward primer | CTGGGCTACACTGAGCACC |
| Reverse primer | AGTGGTCGTTGAGGGCAATG |
| **U6** |  |
| Forward primer | CTCGCTTCGGCAGCACA |
| Reverse primer | AACGCTTCACGAATTTGCGT |
