# Supplementary figures and images for: METTL3 affects FLT3-ITD+ acute myeloid leukemia by mediating autophagy by regulating PSMA3-AS1 stability
Source: Cell Cycle. 2023 Apr 23;22(10):1232–45. doi: 10.1080/15384101.2023.2204770 (PMC10193868; doi:10.1080/15384101.2023.2204770)

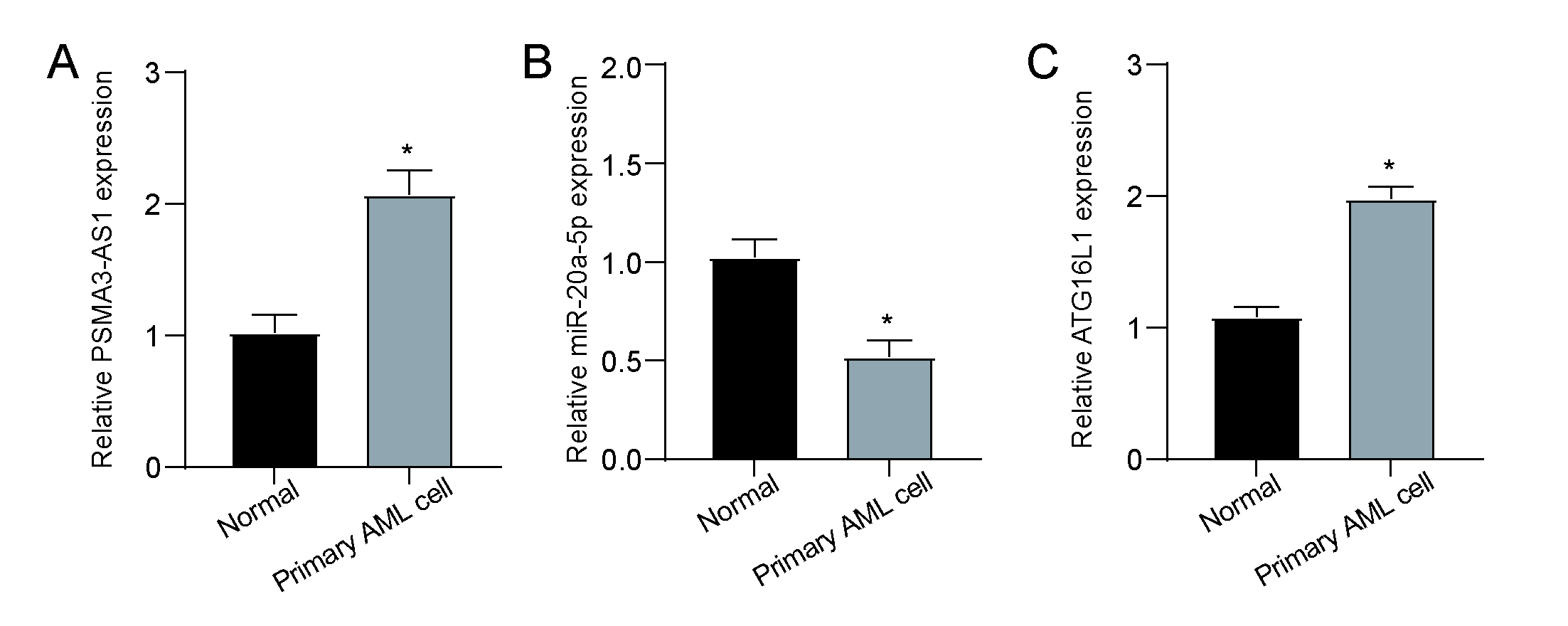

Supplement: Supplemental Material [file KCCY_A_2204770_SM0427.zip › Figure S1 (2).jpg]

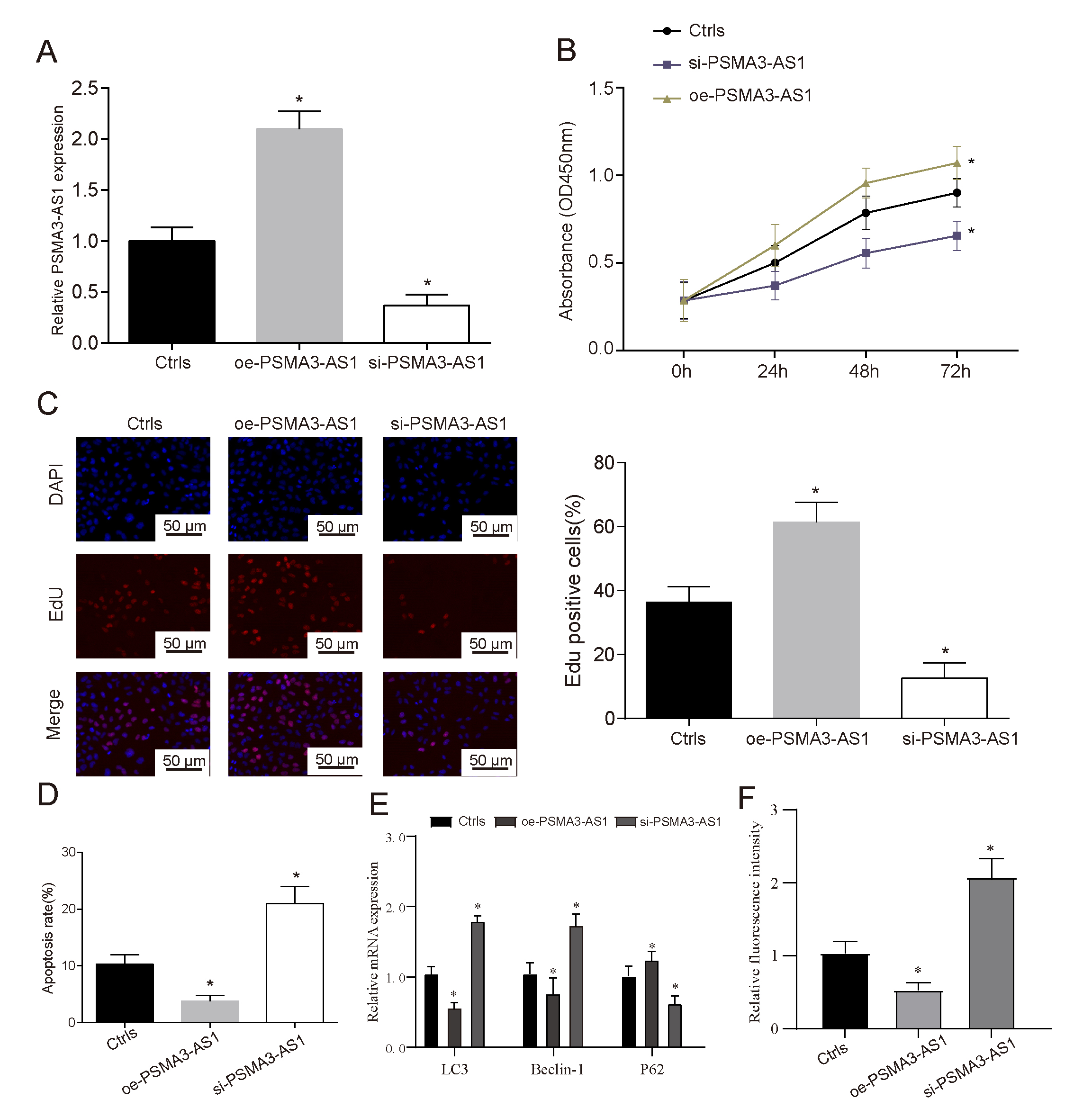

Supplement: Supplemental Material [file KCCY_A_2204770_SM0427.zip › Figure S2 (1).jpg]

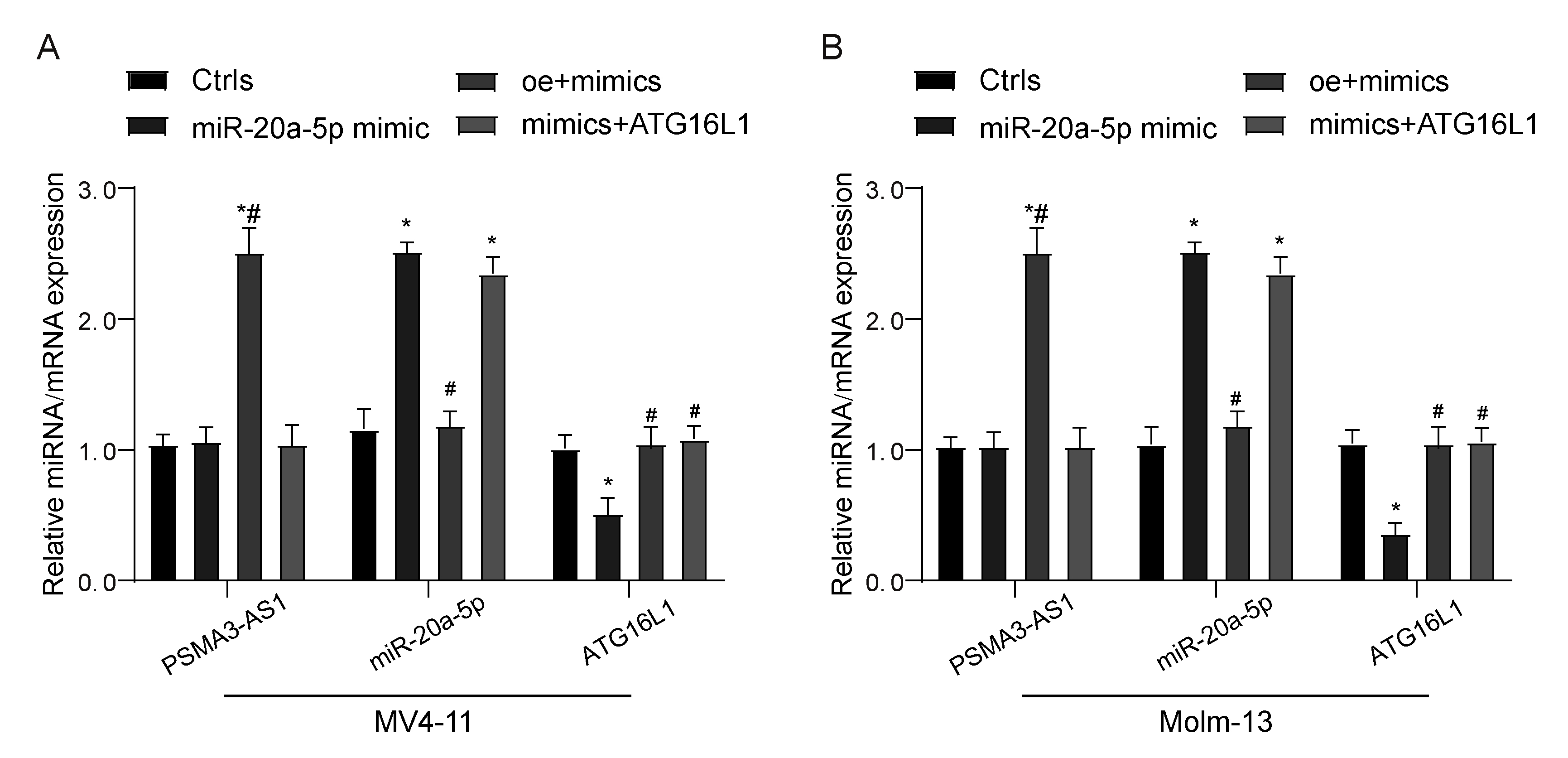

Supplement: Supplemental Material [file KCCY_A_2204770_SM0427.zip › Figure S3 (1).jpg]

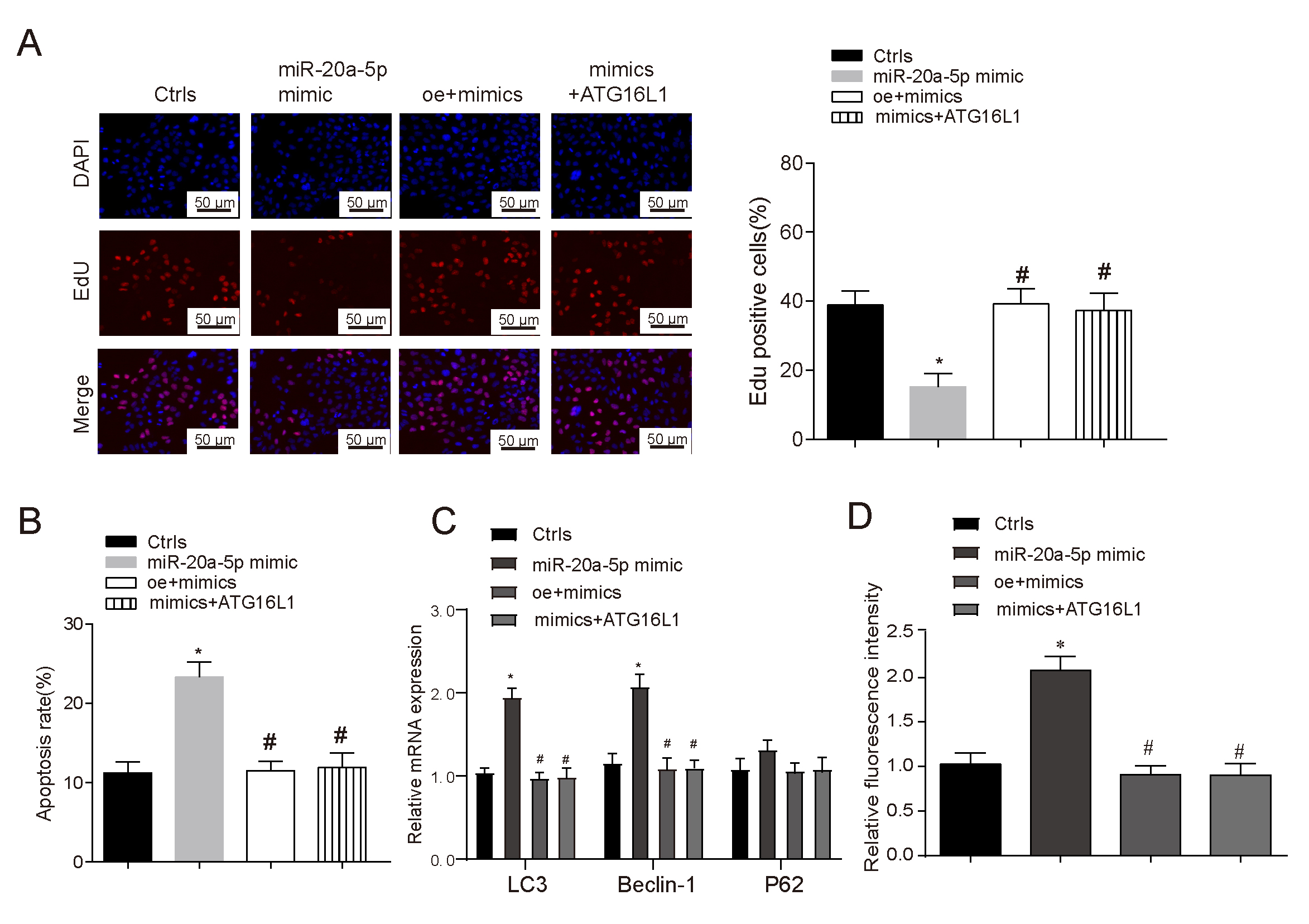

Supplement: Supplemental Material [file KCCY_A_2204770_SM0427.zip › Figure S4.jpg]

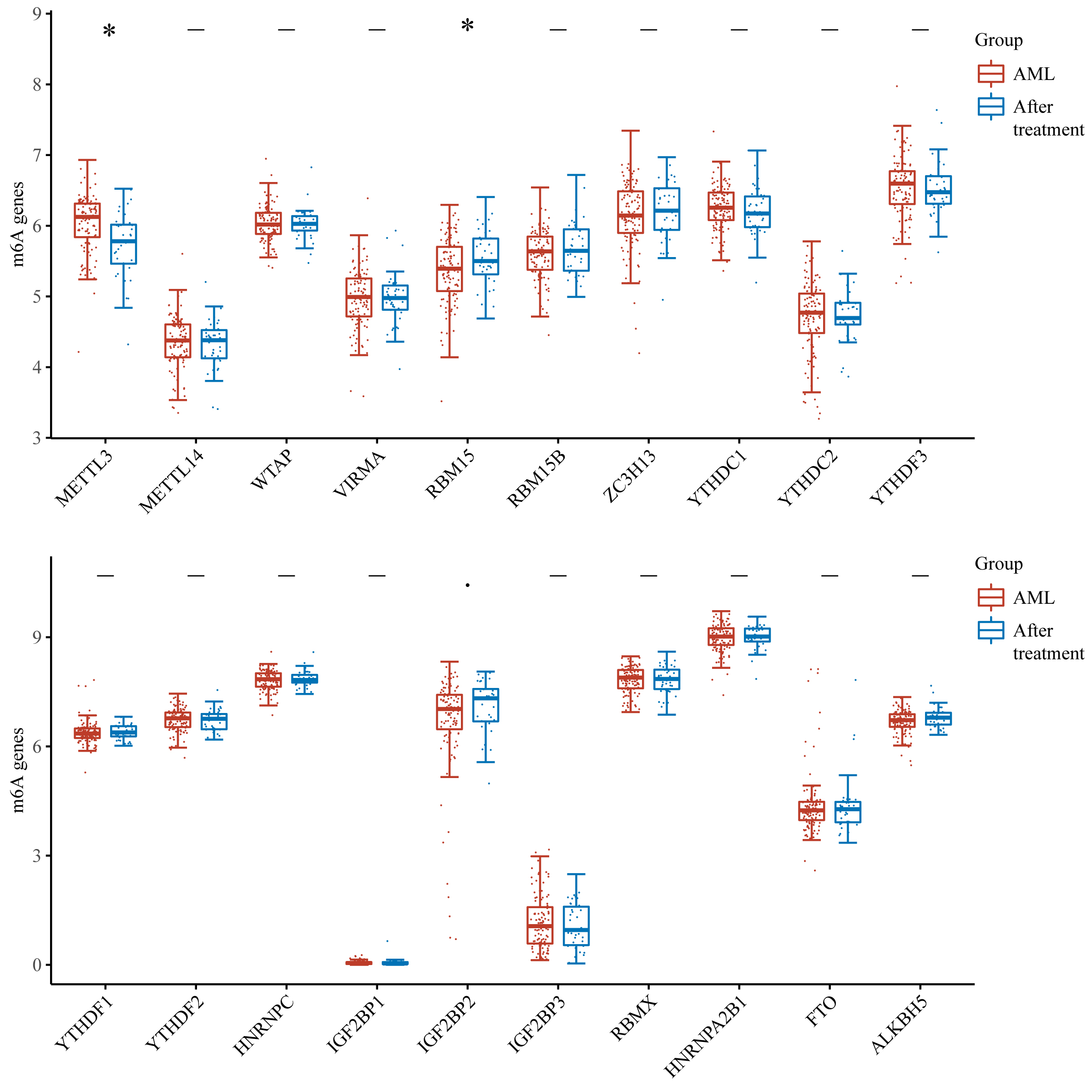

Supplement: Supplemental Material [file KCCY_A_2204770_SM0427.zip › Figure S5.jpg]
